# Congenital Myotonic Dystrophy with Combined Heterozygous ATP8B1/ABCB4 Mutation Leading to Progressive Cholestasis and Liver Failure

**DOI:** 10.1097/PG9.0000000000000121

**Published:** 2021-10-25

**Authors:** Fang Kuan Chiou, Hina Rizvi, Ros Quinlivan, Girish L. Gupte

**Affiliations:** From the * Liver Unit (including small bowel transplantation), Birmingham Women’s and Children’s Hospital NHS Foundation Trust, Birmingham, UK; † Gastroenterology, Hepatology & Nutrition Service, Paediatric Medicine, KK Women’s and Children’s Hospital, Singapore, Singapore; ‡ MRC Centre for Neuromuscular Diseases, National Hospital for Neurology and Neurosurgery, London, UK

**Keywords:** dystrophia myotonica, progressive familial intrahepatic cholestasis, liver transplantation

## Abstract

Myotonic dystrophy (MyoD) is an inherited genetic disorder caused by the expansion of a CTG trinucleotide repeat in the dystrophia myotonica protein kinase gene. It manifests as a multisystem disease affecting not only skeletal muscles, but also heart, lung, eye, gastrointestinal tract, central nervous system, and endocrine system. However, MyoD is rarely associated with a progressive liver disorder. We report a case of congenital MyoD with combined heterozygous ATP8B1/ABCB4 mutation who developed chronic, progressive low gamma-glutamyltransferase cholestatic liver disease at early infancy, and eventually underwent successful liver transplantation.

## BACKGROUND

Myotonic dystrophy (MyoD) is an autosomal dominant, multisystem disorder caused by expansion of CTG trinucleotide repeat in the untranslated region of the dystrophia myotonica protein kinase (*DMPK*) gene on chromosome 19q13.3 (1). There are varying phenotypes and severity of disease that correlates with the size of CTG repeat expansion. In classical, adult-onset MyoD, patients present at the age of 10–30 years with typical features of myotonia, muscle weakness, bulbar insufficiency, cardiorespiratory complications, gastrointestinal (GI) dysmotility, insulin resistance, and cataracts. In contrast, congenital MyoD is the most severe form presenting at birth with respiratory distress, severe hypotonia, and feeding difficulties. If the neonate survives, complications may improve with age but developmental delay, autistic features, and behavioral problems are frequent. Other features include cardiac conduction abnormalities, GI dysmotility, scoliosis, and vesicoureteric reflux. In MyoD, there is an increased risk of anesthetic complications, including life-threatening reactions to muscle relaxants which should be avoided.

Interestingly, while the genetic aberration in MyoD results in multisystem involvement, it is not commonly associated with primary hepatic disease. We describe a child with congenital MyoD, who developed progressive familial intrahepatic cholestasis (PFIC)-like liver disease at early infancy and eventually underwent successful liver transplantation (LT).

## CASE

A newborn with congenital MyoD, diagnosed by a confirmed expansion in the *DMPK* gene in both patient and mother, was delivered at 34 weeks gestation for the indication of reduced fetal movement. Postnatally the child had respiratory distress, hypotonia, and required parenteral nutrition (PN) from day 1 up till 6 months because of GI dysmotility and feed intolerance.

At 2 months, the infant developed conjugated hyperbilirubinemia with pigmented stool and normal hepatobiliary ultrasonographic findings. Screening tests for etiologies of neonatal/infantile cholestasis, including intrauterine infections, alpha-1 antitrypsin deficiency, endocrine, and metabolic disorders were normal.

At 6 months, the infant achieved enteral autonomy with full nasogastric feeds, and PN was discontinued. Despite establishing full feeds with appropriate weight gain, liver dysfunction continued to progress. Serum bilirubin peaked at 471 µmol/L, with alanine transaminase and aspartate transaminase levels at 431 U/L and 648 U/L, respectively. GGT remained low/normal (<50 U/L).

**FIGURE 1.**
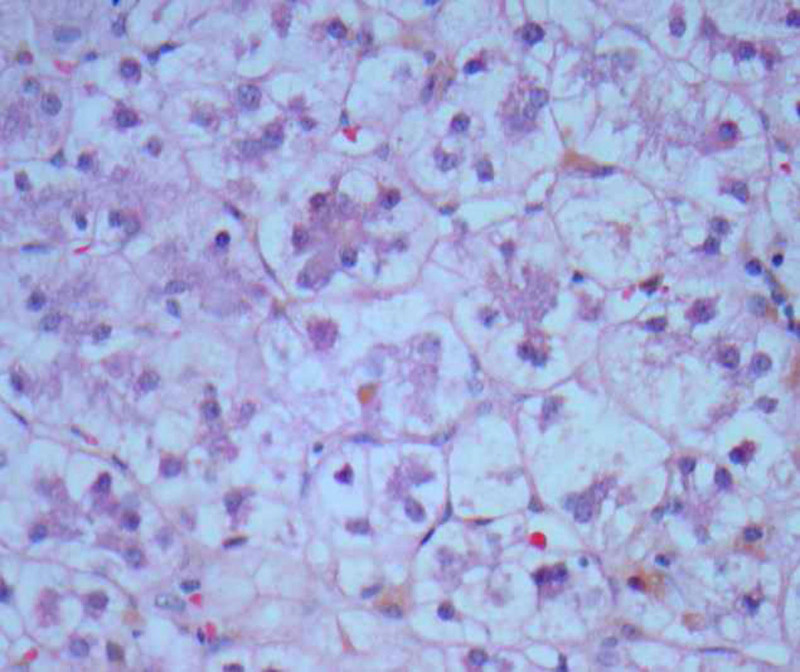
Liver histology at age 10 months, demonstrating cholestasis with scattered canalicular bile plugs, panacinar hepatocyte rosetting, and ballooning (H&E x400).

Liver biopsy at 10 months of age demonstrated histologic features in keeping with liver injury associated with chronic PN (2) (Figure 1). The most significant feature on immunostaining was absent GGT expression throughout most lobules and positive expression of bile salt export pump and multidrug resistance-associated protein 2 (MRP2). Although electron microscopy did not demonstrate pathognomonic Byler bile, the overall immunohistologic and biochemical picture supported the provisional diagnosis of PFIC type-1. However, targeted DNA sequencing with a 24-gene cholestasis panel showed heterozygous mutations in ATP8B1 (2855G>A[p.R952Q]) and ABCB4 (1954A>G[R652G]), which are genes associated with PFIC type-1 and type-3, respectively. No mutation was found in the other genes (ABCB11, TJP2, AKR1D1, HSD3B7, CYP27A1, and BAAT) associated with low-GGT cholestatic liver diseases.

The child eventually developed decompensated liver disease and underwent LT at 4 years 9 months. Histology of the explanted liver showed severe cholestasis and established cirrhosis. No anesthetic complication was encountered during surgery and post-transplant recovery was uneventful. At the most recent assessment at 7 years post-LT, the patient was well and had normal liver biochemistry with normal allograft function (serum bilirubin 25 µmol/L, AST 31 U/L, ALT 27 U/L, albumin 39 g/L, and normal coagulation). Except for right-ankle contracture, the child was ambulating, independent in activities of daily living, and did not require any feeding or respiratory support.

The mother of the patient is aware of this Case Report and gave her permission.

## DISCUSSION

This report describes a case of congenital MyoD, who developed progressive cholestatic liver disease and underwent successful LT. While a definite diagnosis could not be made, the etiology of the child’s liver disease was most probably multifactorial.

The molecular pathogenesis of MyoD is thought to be the result of dysregulated RNA splicing related to the CTG trinucleotide repeat expansion, which leads to the production of abnormal isoforms of intracellular proteins, including chloride channel, insulin receptor, and cardiac troponin. As such, MyoD manifests as a multisystem disease affecting not only skeletal muscles, but also the heart, lung, eye, GI tract, central nervous system, and endocrine system (1). Liver disease is seldom reported as an association with MyoD, although liver enzyme elevation has been reported in patients with MyoD (3), and an association with insulin resistance and nonalcoholic steatohepatitis has been described in the adult-onset phenotype (4).

Intestinal failure-associated liver disease (IFALD) could be a probable cause of liver dysfunction during early infancy. A spectrum of histopathological changes is associated with IFALD, including centrilobular cholestasis, portal inflammation, steatosis, and fibrosis/cirrhosis in more advanced diseases, and these were demonstrated in the first liver biopsy. However, IFALD usually improves with establishing feeds and cessation of PN before the onset of severe fibrosis or cirrhosis (2).

Low GGT and immunohistochemistry findings also raised the possibility of PFIC1, although genetic studies did not confirm the diagnosis. PFIC-like phenotype has been reported in single-allele mutation or combined heterozygous mutations in 2 different PFIC genes. It has been hypothesized that even in a heterozygous state, the mutated protein may have a dominant deleterious effect leading to overall impaired protein expression/function (5). Moreover, other modifier genes and environmental factors could cumulatively contribute to liver dysfunction and chronic liver disease.

We postulate that the early progressive liver disease, in this case, was the result of cumulative injury arising from multiple primary and secondary insults. First, we postulate that congenital MyoD may be associated with a predisposition to subclinical hepatic dysfunction (6) given that the genetic disorder is known to affect multiple systems. Second, low GGT expression supports the hypothesis that the dual effects of heterozygous mutations in both *ATP8B1* and *ABCB4* genes could have resulted in the “PFIC-like” phenotype in this patient. Heterozygous 2855G>A[p.R952Q] mutation in the *ATP8B1* gene has been described in benign recurrent intrahepatic cholestasis and intrahepatic cholestasis of pregnancy (7,8), whereas *ABCB4* mutation, 1954A>G[R652G], has been reported to be pathogenic for PFIC type-3 (9). Third and very importantly, IFALD was a key factor that triggered liver dysfunction at the initial stage. These additive injuries led to chronic, progressive liver disease, and eventual hepatic decompensation.

In considering a MyoD patient for LT, disease severity, organ involvement, and overall quality of life should be taken into account. Our patient did not have cardiorespiratory involvement and underwent successful LT surgery with no major surgical or anesthetic complications. Specific anesthetic recommendations exist for patients with MyoD, as these patients are at increased risk of cardiac, respiratory, and gastrointestinal complications especially in the postoperative period (10).

We acknowledge that whole-exome sequencing may be useful in detecting potential rare/unknown mutation, but this was not performed as the patient’s liver disease was functionally cured after LT

In summary, we describe an uncommon association between congenital MyoD and predisposition to progressive liver disease which can be successfully managed with LT.
